# Cellular memory of hypoxia elicits neuroblastoma metastasis and enables invasion by non-aggressive neighbouring cells

**DOI:** 10.1038/oncsis.2014.52

**Published:** 2015-02-09

**Authors:** A Herrmann, M Rice, R Lévy, B L Pizer, P D Losty, D Moss, V Sée

**Affiliations:** 1 Department of Biochemistry, University of Liverpool, Liverpool, UK; 2 Department of Cellular and Molecular Physiology, University of Liverpool, Liverpool, UK; 3 Department of Paediatric Oncology Alder Hey Children's NHS Foundation Trust, Liverpool, UK; 4 Academic Paediatric Surgery, Division of Child Health, University of Liverpool, Liverpool, UK

## Abstract

Therapies targeting cancer metastasis are challenging owing to the complexity of the metastatic process and the high number of effectors involved. Although tumour hypoxia has previously been associated with increased aggressiveness as well as resistance to radio- and chemotherapy, the understanding of a direct link between the level and duration of hypoxia and the individual steps involved in metastasis is still missing. Using live imaging in a chick embryo model, we have demonstrated that the exposure of neuroblastoma cells to 1% oxygen for 3 days was capable of (1) enabling cell migration towards blood vessels, (2) slowing down their velocity within blood vessels to facilitate extravasation and (3) promoting cell proliferation in primary and secondary sites. We have shown that cells do not have to be hypoxic anymore to exhibit these acquired capabilities as a long-term memory of prior hypoxic exposure is kept. Furthermore, non-hypoxic cells can be influenced by neighbouring hypoxic preconditioned cells and be entrained in the metastatic progression. The acquired aggressive phenotype relies on hypoxia-inducible factor (HIF)-dependent transcription of a number of genes involved in metastasis and can be impaired by HIF inhibition. Altogether, our results demonstrate the need to consider both temporal and spatial tumour heterogeneity because cells can 'remember' an earlier environment and share their acquired phenotype with their close neighbours. As a consequence, it is necessary to monitor the correct hypoxic markers to be able to predict the consequences of the cells' history on their behaviour and their potential response to therapies.

## Introduction

During tumour progression, cells can become invasive and colonise distant organs.^1^ This phenomenon called metastasis is a critical problem contributing to more than 90% of cancer deaths^2^ and involves several steps from the initial detachment from the primary site, diffusion within the surrounding tissue, intravasation into the blood stream to extravasation and proliferation in the metastatic site to form secondary tumours.

Increased tumour progression and metastasis have previously been associated with poorly oxygenated (hypoxic) regions in primary tumours.^3, 4, 5^ Neuroblastoma (NB), a highly malignant paediatric solid tumour developing from neural-crest-derived cells during foetal or early postnatal life, is one example in which a hypoxic signature is associated with dismal patient outcome.^6^ Hypoxia is known to trigger dedifferentiation of NB cells towards a more immature stem cell phenotype and is associated with NB metastasis.^7^ More than 60% of NB tumours are metastatic and secondary tumours can be found in bone, bone marrow, liver, lymph nodes or, less commonly, in the skin, lung or brain.^8^

As the oxygen levels found in solid tumours are dynamic, with cycles of hypoxia and reoxygenation^9^ and heterogeneous within the tumour,^10, 11^ it is not trivial to demonstrate a direct link between oxygen levels and the molecular mechanisms associated with metastasis. For example, it is unknown which level and duration of hypoxic exposure is necessary to induce long-lasting changes in cancer cells leading to the emergence of an aggressive and metastatic phenotype. To visualise directly the effects of hypoxic preconditioning from primary tumour invasion into blood vessels to secondary tumour formation in distant organs sites, we have used a chick embryo model combined with live *in vivo* and *ex vivo* imaging.

Initially shown to support murine sarcoma xenografts, the chick embryo has been exploited for more than 100 years.^12^ The chorioallantoic membrane (CAM) is a well-vascularised extraembryonic tissue located underneath the eggshell and constitutes an excellent biological platform for the molecular analysis of cancer including xenografting, angiogenesis and metastasis.^13^ As the chick embryo is naturally immunodeficient in the early stages of development,^14^ the CAM readily supports the engraftment of tumour tissues.

We have shown here that NB cells can be successfully grown as a tumour on the CAM, without spontaneous dissemination. We further demonstrate that hypoxic preconditioning modifies the cells' phenotype, leading to metastasis into the chick organs. We report that hypoxic preconditioning affects cell adhesion in opposing ways depending on the metastatic stage and triggers durable changes in gene expression *in vivo.* Importantly, we have observed that cells preexposed to a hypoxic environment can trigger metastasis of non-hypoxic cells located in close proximity, which would not have metastasised otherwise. These observations of spatial (entrainment) and temporal (long-term changes) coupling provide a well-grounded experimental basis towards the construction of spatiotemporal models of tumour development, which will be essential to understand the metastatic potential of a cell.

## Results

### Hypoxia induces invasion and metastasis *in vivo*

**Figure 1:**
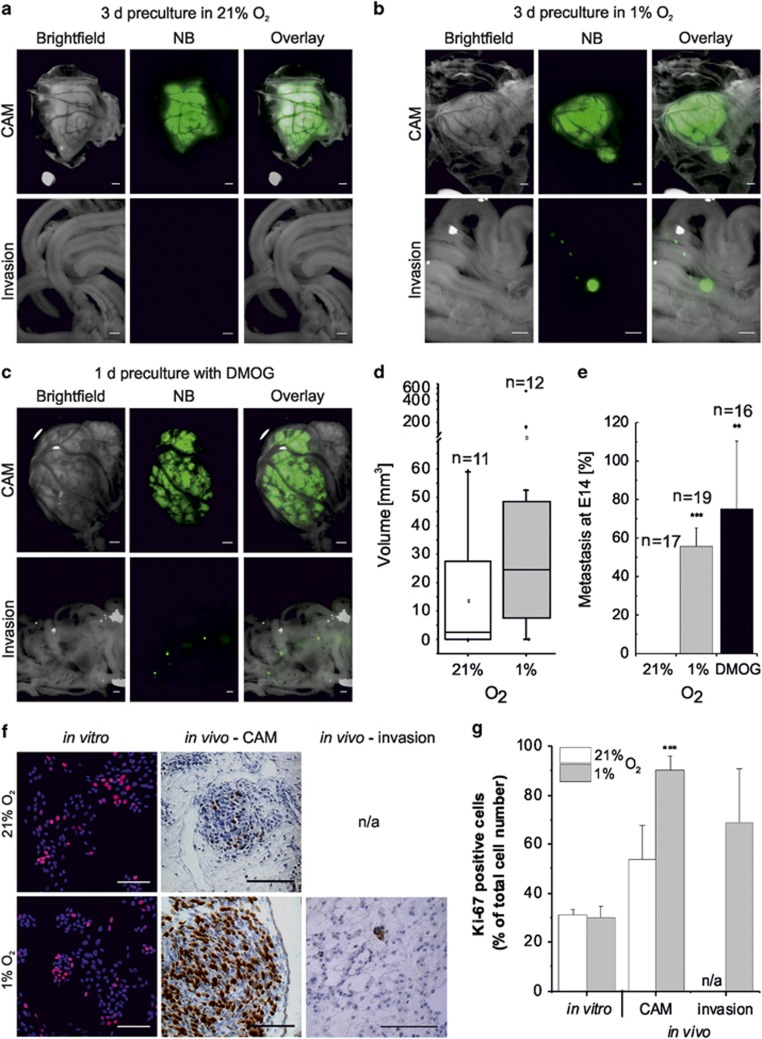
Preculture in 1% O_2_ for 3 days triggers metastasis *in vivo.* EGFP-labelled SK-N-AS cells were implanted on the CAM of E7 chick embryos. Tumourigenesis on the CAM and metastasis inside the chick organs was monitored at E14. (**a**) Representative images of tumourigenesis (upper panel) and invasive behaviour in the intestine (lower panel) are shown for cells grown under normoxia. Scale bar is 500 μm. (**b**) Same as in (**a**) with cells precultured for 3 days in 1% O_2_, before CAM implantation. (**c**) Same as in (**a**) with SK-N-AS cells pretreated with DMOG (0.5 mm, 1 day). (**d**) Box plot depicting mean volume of tumours formed on the CAM at E14 by SK-N-AS cells precultured at 21% (white, *n*=11) or 1% O_2_ (grey, *n*=12) for 3 days. (**e**) Quantitative analysis of metastatic tumour occurrence measured at E14. Displayed is the percentage of embryos with metastasis observed in any site relative to tumour-bearing chicks. Bars represent the mean±s.e.m. from at least 16 embryos. **P⩽0.01 and ***P⩽0.001 compared with normoxia. (**f**) Ki-67 staining (red for *in vitro* and brown for *in vivo*) of SK-N-AS cells cultured for 3 days at 21% O_2_ or 1% O_2_ cultured *in vitro* (nuclei (blue) staining with Hoechst 33342) or *in vivo* forming primary tumours on the CAM or secondary tumours in chick embryos. Scale bar is 100 μm. (**g**) Quantification of Ki-67-positive cells out of the total cell number. Bars represent mean±s.e.m. from three independent experiments. ***P⩽0.001 compared with normoxia.

Hypoxic tumour signature is usually monitored by the levels of hypoxia-inducible factor 1α or 2α (HIF-1α or HIF-2α) or by the expression of their target genes (for example, carbonic anhydrase IX (CA9)).^15^ While the correlation between a hypoxic signature and poor clinical prognosis is accepted for several tumours, including NB,^6, 16, 17^ the duration and levels of hypoxia responsible for this association remain unknown. To investigate how hypoxia affects the growth and aggressiveness of NB, we grew NB cells on the CAM of the chick embryo. Fluorescently labelled SK-N-AS cells, which are derived from a metastatic site (bone marrow), were cultured under normoxia, hypoxia (3 days in 1% O_2_) or treated with the hypoxia mimic dimethyloxaloylglycine (DMOG). They were implanted on the CAM at E7 and their ability to form tumours and to invade chick tissues was assessed. Cells grown in normoxia were capable of tumourigenesis but not of metastatic invasion (Figure 1a). Although the primary tumour morphology was variable, all tumours were found to be heavily vascularised. Some tumours also presented as haemorrhagic lesions or surrounded by haematoma (Supplementary Figure S1A), a morphology commonly present in NB.^18^ Interestingly, SK-N-AS cell preconditioning in 1% O_2_ for 3 days led not only to tumourigenesis on the CAM but also to clear metastasis in the chick embryo organs (Figure 1b). Frequent sites of secondary tumour formation were the gut, adjoining tissues (mesentery) and, crucially, the liver, which is a common site for tumour metastasis in patients with NB.^8, 19^ Other less frequently invaded regions were the kidney and meninges (gallery of images in Supplementary Figure S1B).

Hypoxia primarily triggers HIF stabilisation, due to a decrease of the activity of the prolyl-hydroxylase domain enzymes. To investigate if the hypoxia-driven metastasis was due to a decrease in prolyl-hydroxylase domain activity, cells were treated for 1 day with DMOG, a reversible prolyl-hydroxylase domain inhibitor causing HIF-1/2α accumulation regardless of external oxygen tension. Similar to hypoxia, DMOG treatment resulted in both tumourigenesis and invasion (Figure 1c), indicating a likely role of HIF in the induction of NB metastasis.

Primary tumour occurrence was similar among the conditions (normoxia: 40% hypoxia: 56% DMOG: 58%); however, tumours formed by cells precultured in hypoxia grew to a larger volume than those of cells precultured in normoxia, with a mean of 76 and 13 mm^3^, respectively (Figure 1d). More importantly, cells cultured in normoxia did not show any metastatic potential (0%), compared with 52% and 75% for preconditioned cells in 1% O_2_ or DMOG, respectively (Figure 1e). We further examined if hypoxic preconditioning influenced cell proliferation (Figure 1f). Exposure of NB cells to 1% O_2_ for 3 days had no effect on the number of proliferative cells *in vitro*, with an average of 30% Ki-67-positive cells in both conditions. *In vivo*, however, hypoxic preconditioning caused an increase in proliferation on the CAM from 54 to 89% positive cells, consistent with the larger tumour volume observed, and 69% proliferation in metastasised cells (Figure 1g).

Amplification of the proto-oncogene transcription factor MYCN is found in about 25% of NB cases and correlates with high-risk disease and poor prognosis.^20^ As SK-N-AS cells are non-MYCN-amplified, the same experiments were repeated with the MYCN-amplified SK-N-BE(2)C cell line. Similar results were obtained (Supplementary Figures S1C–E), indicating that the impact of hypoxia on NB metastasis is likely to be independent of the MYCN amplification.

### Invasion by non-metastatic cells can be triggered by close contact with metastatic cells

The pathological low oxygen tension found in most solid tumours is described as heterogeneous and dynamic in space and time.^10^ It is therefore expected that a mixed population of hypoxic and normoxic cells coexists as opposed to one homogenous cell population. To reproduce a more pathophysiological situation, and to assess if the presence of a small number of hypoxic cells could influence normoxic non-metastatic cells, we implanted mixed-cell populations. Cells were labelled with either enhanced green fluorescent protein (EGFP) or dTomato. To exclude the possibility that tumour cell behaviour is influenced by the fluorescent protein used to label the cells, all experiments conducted with EGFP-expressing cells (Figure 1) were reproduced with dTomato-expressing cells leading to similar results (Supplementary Figures S2A and B).

**Figure 2:**
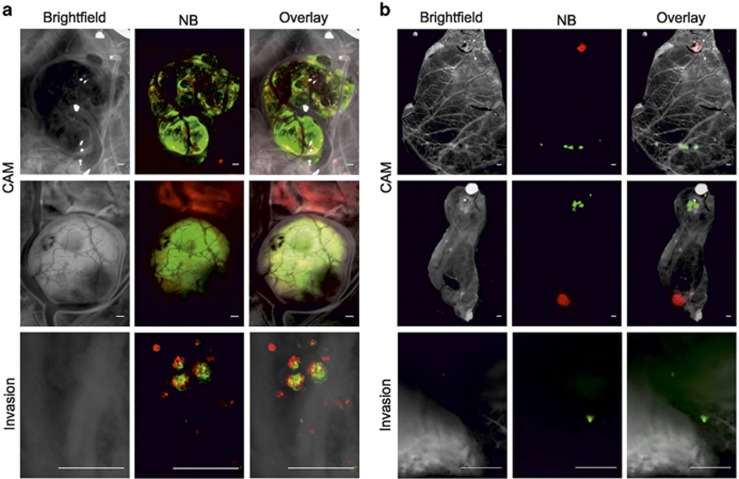
Invasion of normoxic cells can be initiated by direct interaction with hypoxic cells. EGFP- and dTomato-labelled SK-N-AS cells were implanted on the CAM of E7 chick embryos. SK-N-AS-dTomato cells were precultured at 21% O_2_ for 3 days and SK-N-AS-EGFP cells were precultured at 1% O_2_ for 3 days. (**a**) SK-N-AS-EGFP and SK-N-AS-dTomato cells were mixed before CAM implantation. Pictures show tumourigenesis on the CAM (upper panel) and invasive behaviour in the intestine (lower panel) at E14. Scale bar is 500 μm. (**b**) SK-N-AS-EGFP and SK-N-AS-dTomato cells were implanted separately on different locations on the CAM. Pictures show tumourigenesis on the CAM at E14 and intestinal metastasis by the SK-N-AS-EGFP. Scale bar is 500 μm.

First, we implanted a mixed population of SK-N-AS-dTomato cells precultured in normoxia and SK-N-AS-EGFP cells precultured for 3 days in 1% O_2_. Tumourigenesis took place as described previously. Interestingly, metastasis occurred not only for cells precultured in hypoxia but also for the normoxic ones (Figure 2a, metastasis in the intestine and mesentery). We then investigated if a close but indirect contact of hypoxic and non-hypoxic cells could impact on their invasive ability. Hypoxic and normoxic preconditioned cells were implanted on different locations on the CAM of the same chick embryo. Again, tumourigenesis was observed for both cell types, with the tumours being physically separated and no obvious cell mixing (Figure 2b). In this case, the metastatic dissemination was only detected for cells precultured in hypoxia (Figure 2b), indicating that hypoxic preconditioning enables invasion of cells unable to metastasise on their own; however, close proximity and direct cell–cell contact are needed for such facilitation.

### Hypoxia promotes active intravasation and slows down the vascular migration

**Figure 3:**
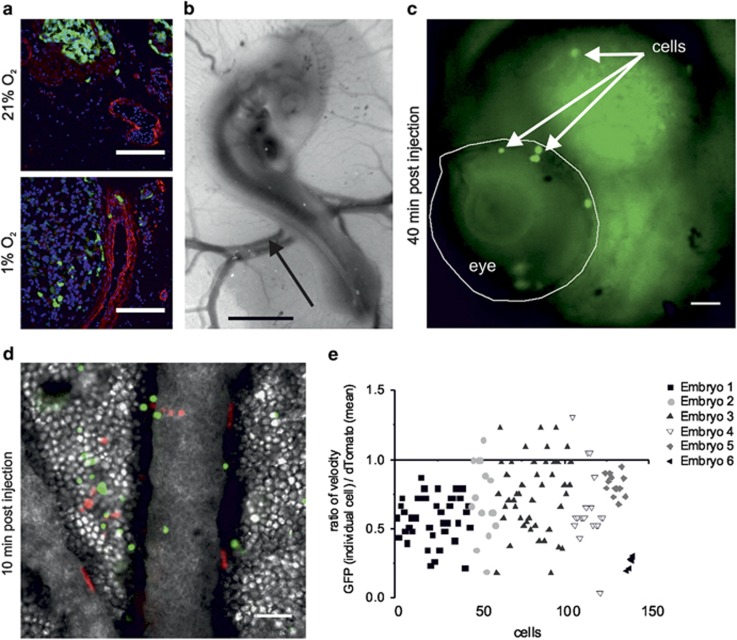
Hypoxia promotes active intravasation and slows vascular migration. (**a**) Representative pictures of CAM tumour sections from tumours formed at E14 by SK-N-AS-EGFP cells precultured in 21% O_2_ (upper panel) or 1% O_2_ (lower panel) for 3 days. SK-N-AS cell (green) proximity to the blood vessels (smooth muscle actin staining, red) is observed for hypoxic but not normoxic preconditioned cells. Nuclei are stained with Hoechst 33342 (blue). Scale bar is 100 μm. (**b**) Representative image of a chick embryo at E3, with its extraembryonic blood vessels. The arrow indicates the site of intravenous injection. Scale bar is 500 μm. (**c**) Typical image from live *ex ovo* imaging 40 min after injection of SK-N-AS-EGFP cells in an E3 chick embryo. The eye of the embryo is outlined. The arrows point to some EGFP-labelled SK-N-AS cells settled in the embryo. Scale bar is 20 μm. (**d**) SK-N-AS-EGFP (precultured in 1% O_2_, 3 days) and dTomato cells (precultured in 21% O_2_, 3 days) were coinjected in the blood vessels of an E3 chick embryo as shown in (**c**). The picture was taken 10 min after injection by live *ex ovo* imaging. Image capture time is 500 ms and scale bar is 100 μm. (**e**) Velocity of coinjected SK-N-AS cells precultured at 21% O_2_ or 1% O_2_ was calculated as indicated in Materials and methods. Approximately 150 cells for each condition acquired from six embryos were quantified. The plot represents the ratio of velocity for each individual EGFP cells divided by the average velocity of the dTomato cells. The mean ratio 0.66 is statistically different from 1 with *P*⩽0.001. The absolute mean velocity was measured as 1600 μm/s for cells preconditioned in hypoxia and 3200 μm/s for normoxic cells.

We then investigated which steps of the metastatic process were enabled by hypoxic preconditioning. We first assessed if hypoxic preconditioning had an effect on how cells could migrate towards blood vessels by staining CAM tumour samples with a marker of the smooth muscle cells. Cells were detected at or within the vasculature only for tumours formed by hypoxic preconditioned cells (Figure 3a), indicating that hypoxia facilitates and potentially even enables the intravasation of cells into the circulation.

To assess if hypoxia also influences margination (attachment to the endothelial layer of the vasculature), we directly injected cells intravenously at E3 (Figure 3b). Chick embryos were then imaged live, *ex ovo*, for up to 30 h after injection. At 40 min after injection, some SK-N-AS cells had already settled in tissues, whereas others were still circulating in the vasculature (Figure 3c and Supplementary Video S1). To quantify cell migration within blood vessels, a single-cell suspension of EGFP- (hypoxic preconditioning) and dTomato-labelled SK-N-AS cells (normoxic preconditioning) was coinjected and cell velocity in the blood vessels was measured using fast fluorescence imaging (Figure 3d and Supplementary Video S2). Cells preconditioned in hypoxia moved, on average, at half the speed of the cells preconditioned in normoxia (Figure 3e). This will likely facilitate cell attachment to the vessel wall and subsequently enable extravasation. Altogether, these data indicate that, while hypoxia enhances cell migration from the primary tumour towards blood vessels, it also clearly decreases the velocity within the vasculature. In this respect, the changes in cell adhesion properties triggered by hypoxia are likely to be multifaceted.

### Hypoxic preculture increases invasion, microtumour formation and is dependent of duration and level of hypoxia

**Figure 4:**
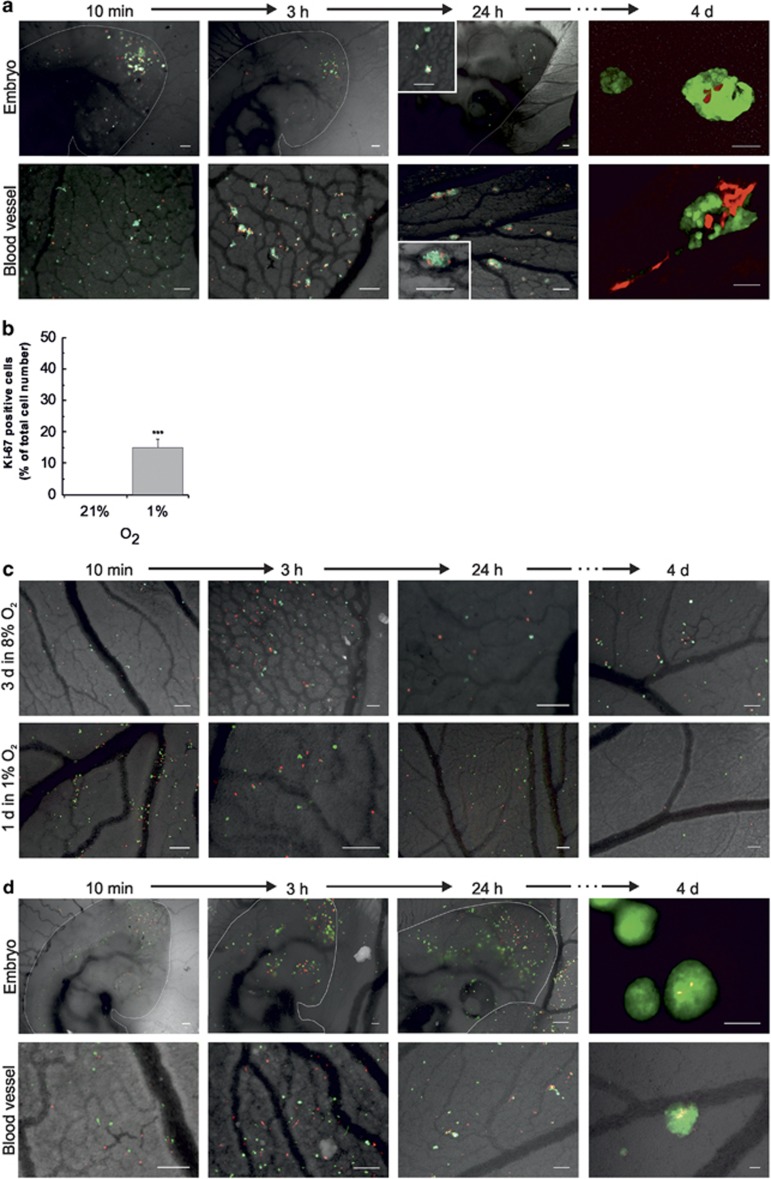
Hypoxia-induced microtumour formation is dependent on the duration and level of oxygen preconditioning. (**a**) SK-N-AS-dTomato (precultured in 21% O_2_, 3 days) and SK-N-AS-EGFP cells (precultured in 1% O_2_, 3 days) were coinjected intravenously in E3 chick embryos. Microtumour formation was followed over time. The pictures show SK-N-AS cells in the chick embryo (upper panel) or its extraembryonic blood vessels (lower panel), 10 min, 3 h, 24 h and 4 days after intravenous coinjection. Scale bar is 200 μm for 10 min, 3 h, 24 h and 50 μm for 4-day images. (**b**) Quantification of Ki-67-positive cells relative to total cell number in metastatic sites, and 7 days after intravenous injection. Bars represent mean±s.e.m. from three independent experiments. ***P⩽0.001 compared with normoxia. (**c**) Microtumour formation does not occur in mild hypoxia (8% O_2_) or a shorter exposure to 1% O_2_. SK-N-AS-dTomato (precultured in 21% O_2_, 3 days) were coinjected with SK-N-AS-EGFP preconditioned for 3 days in 8% O_2_ (upper panel) or for 1 day in 1% O_2_ (lower panel). Images show SK-N-AS cells in the extraembryonic blood vessels, 10 min, 3 h, 24 h and 4 days after intravenous injection. Scale bar is 200 μm. (**d**) SK-N-AS-EGFP (pretreated with 0.5 mm DMOG for 1 day) and dTomato cells (precultured in 21% O_2_, 1 day) were coinjected intravenously in E3 chick embryos. DMOG treatment results in microtumour formation over time. The pictures show SK-N-AS cells in the chick embryo (upper panel) or its extraembryonic blood vessels (lower panel), 10 min, 3 h, 24 h and 4 days after intravenous coinjection. Scale bar is 200 μm.

We further sought to elucidate the long-term consequences of the different vascular migration pattern, by monitoring the coinjected cells over time, *in ovo*, up to E10. In the chick embryo, as well as in its extraembryonic blood vessels, hypoxic preconditioned cells formed aggregates, as early as 10 min after injection, whereas cells precultured in normoxia were rather found as single, isolated cells (Figure 4a). The initial aggregates formed by hypoxic cells turned into rounded microtumours within 24 h. To assess proliferation, samples of coinjected cells were stained for Ki-67. At E10, the few remaining cells from normoxic preculture failed to display any positive staining, whereas 15% of the cells preexposed to hypoxia were proliferative (Figure 4b). This is consistent with results previously obtained in the CAM model (Figure 1g).

To elucidate the oxygen level and exposure time necessary to induce the observed invasive phenotype, SK-N-AS-dTomato cells cultured in normoxia were coinjected with SK-N-AS-EGFP cells cultured in either 8% or 1% O_2_ for 3 days or 1 day, or pretreated with DMOG as a comparison. Preconditioning at physiological normoxia (8% O_2_ for 3 days) failed to induce microtumour formation as well as a short-term exposure of 1 day in 1% O_2_ (Figure 4c). In contrast, DMOG-treated cells formed microtumours (Figure 4d). In summary, hypoxic preconditioning needs to consist of at least 3 days at 1% O_2_ to promote extravasation and tumour formation in secondary sites.

### Hypoxic preconditioning is sufficient to activate long-lasting transcription of several prometastatic genes

**Figure 5:**
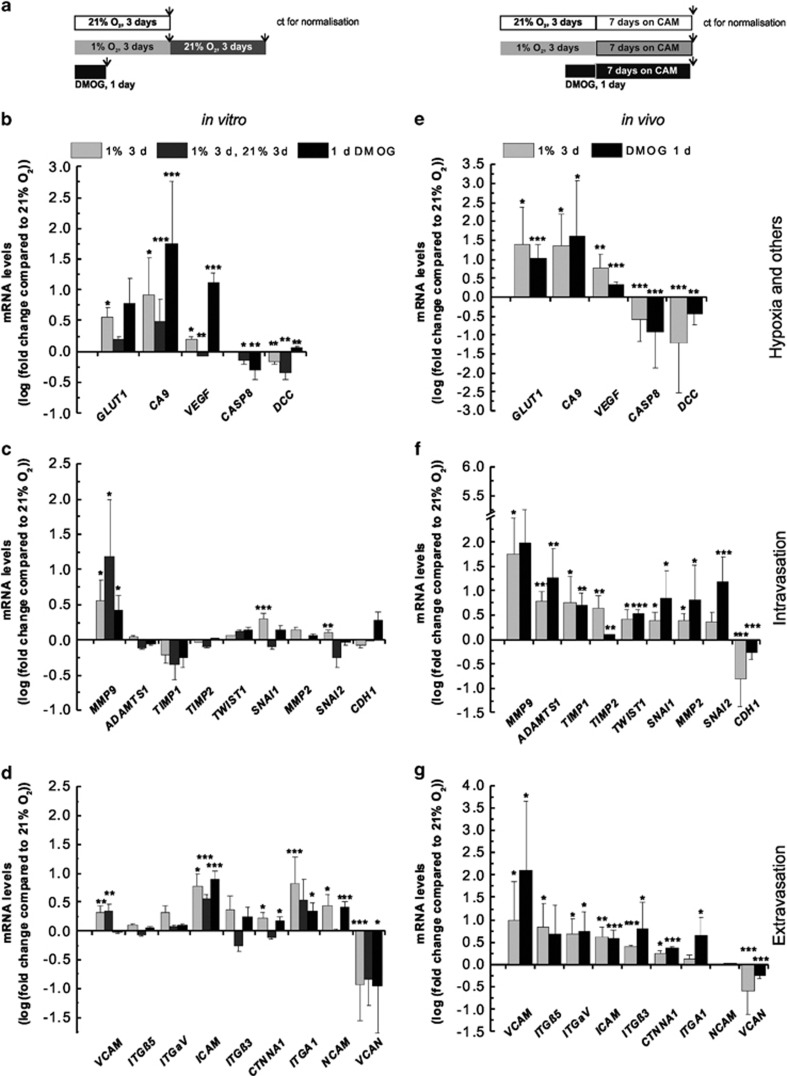
Hypoxic exposure triggers long-lasting changes in the expression of genes involved in invasion, intravasation, adhesion and extravasation. (**a**) Schematic representation of the experimental protocol. Arrows indicate when samples were collected for qPCR analysis. Relative mRNA levels of genes related to hypoxia and poor prognosis (**b** and **e**), invasion and intravasation (**c** and **f**) and adhesion and extravasation (**d** and **g**) were measured by qPCR in cells cultured *in vitro* (**b**–**d**) and in cells forming tumours (**e**–**g**). (**b**–**d**) SK-N-AS cells were cultured for 3 days in 21% O_2_ (control for normalisation), 1% O_2_ (grey bar), 3 days in 1% O_2_, followed by 3 days in 21% O_2_ (dark grey bar) or treated for 1 day with DMOG (black bar). mRNA levels were measured relative to cyclophilin A and normalised to relative levels of cells precultured at 21% O_2_. (**e**–**g**) Cells were precultured for 3 days in 21% O_2_ (control for normalisation), 1% O_2_ (grey bar) or treated for 1 day with DMOG (black bar) before implantation on the CAM at E7. At least six tumours were collected at E14 for each condition and relative mRNA levels are displayed relative to cyclophilin A and normalised to tumours formed with cells precultured for 3 days at 21%. Bars represent the logarithm of the normalised mean±s.e.m. of at least three independent experiments. *P⩽0.05, **P⩽0.01 and ***P⩽0.001 compared with normoxia.

Although we observed profound phenotypic effects triggered by preculture in a hypoxic environment, from the implantation/injection time until the endpoint measurements up to 1 week later, the cells were no longer in a controlled hypoxic environment. Thus, one question was: how long lasting are the effects of a hypoxic preincubation upon reoxygenation? We measured transcriptional outputs by quantitative PCR (qPCR), for a number of genes involved in intravasation (for example, cell–cell adhesion; matrix metalloproteases; epithelial–mesenchymal transition), extravasation (for example, epithelial cell adhesion; glycoproteins) and a classical hypoxic response (for example, vascular endothelial growth factor (*VEGF*), glucose transporter type 1 (*GLUT1*)). For a complete list of the 23 genes measured, their function and heatmap response to hypoxia (see Supplementary Figure S3A). Experimental conditions are detailed in Figure 5a. Figures 5b and d show the relative gene expression of SK-N-AS cells *in vitro*, cultured in hypoxic conditions with or without reoxygenation and normalised to normoxic-untreated conditions.

Interestingly, while the classical hypoxic target genes were sensitive to reoxygenation, with a significant loss of expression of *GLUT1*, *CA9* and *VEGF* after 3 days of reoxygenation (Figure 5b), for several genes involved in the intravasation process, the reoxygenation amplified the regulatory effects initially triggered by hypoxia. For example, for matrix metallopeptidase 9 (*MMP9*), ~15-fold upregulation was observed when hypoxia was followed by reoxygenation compared with ~3-fold increase for hypoxia only (Figure 5c). The response of genes involved in extravasation was regulated by hypoxia and insensitive to reoxygenation (Figure 5d). However, *ITGB5* (integrin-β5), *MMP2* and *NCAM* (neural cell adhesion molecule) were sensitive to reoxygenation, akin to the classical hypoxic target genes. Nevertheless, these results show that a significant number of metastatic genes regulated by hypoxic preconditioning were still up- or downregulated 3 days after reoxygenation and could explain the invasive phenotype observed *in vivo*.

We next measured how the expression of the same genes was regulated in cells forming tumours *in vivo*. Figure 5e, f and g show that 21 out of the 23 genes measured were significantly regulated in the tumours formed by hypoxic or DMOG-treated cells compared with tumours formed by normoxic cells. In comparison, only 13 genes were significantly regulated in cells maintained *in vitro*. Importantly, for several genes, the effects of hypoxia were much stronger. For example, *MMP9* was upregulated more than 50 times in hypoxic tumours compared with the normoxic tumours, but was only increased 3.5 times by hypoxia *in vitro*. This enhancement of gene expression regulation *in vivo* was observed for all genes measured regardless of their up- or downregulation profile. These observations reinforce the concern that cells in culture behave differently from cells in a more physiological and 3D environment and that some molecular changes might be missed in *in vitro* cultures, where extracellular matrix components and tight cell–cell junctions are absent.

Taken together, these results demonstrate that hypoxic preexposure induces a strong regulation of many genes involved in metastasis even 7 days later. The hypoxic memory effect observed in *in vivo* tumours was also observed at the protein level. For example, CA9 staining was markedly pronounced in tumours formed by cells precultured in hypoxia (Supplementary Figure S3B). Surprisingly, staining for HIF-1α and HIF-2α in tumours did not vary between normoxic and hypoxic preincubation (Supplementary Figure S3B), indicating that HIF-1/2α levels might not be the most adequate markers to detect previous hypoxic episodes experienced by tumour cells. The low HIF-2α levels were in agreement with a previous report where SK-N-AS cells, and other NB cell lines, failed to display detectable HIF-2α expression regardless of oxygen tension.^17^

### HIF activity is required for the acquisition of the metastatic phenotype

**Figure 6:**
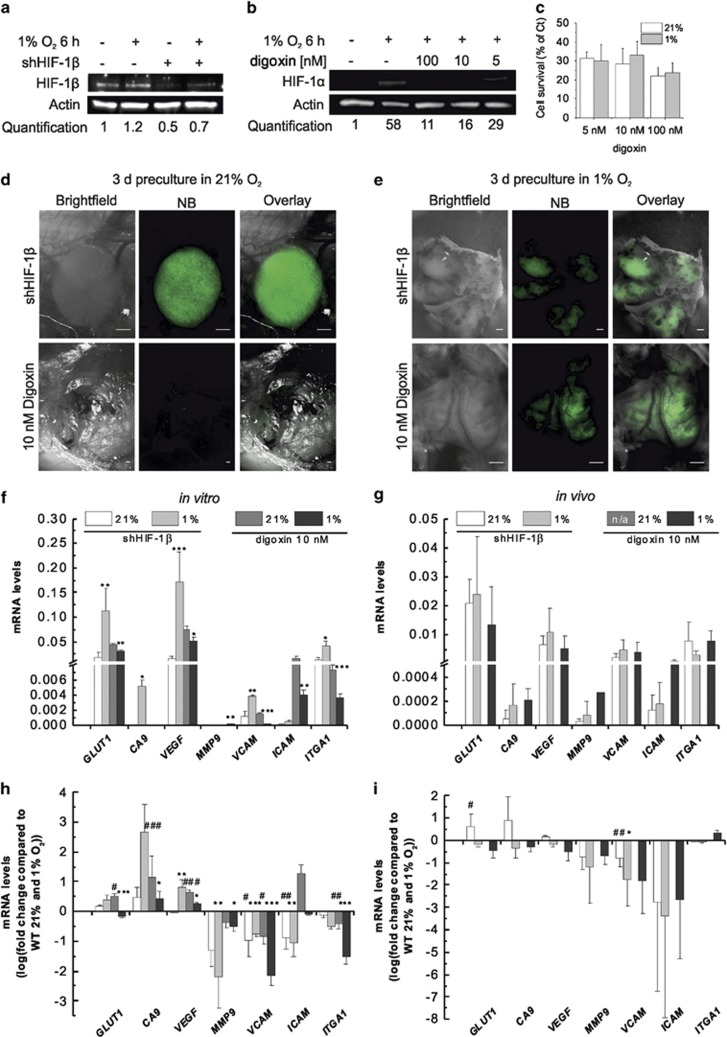
HIF inhibition prevents hypoxia-induced metastasis. (**a**) Validation of HIF-1β knockdown by western blot. SK-N-AS-EGFP cells were stably transduced with shHIF-1β lentivirus. The knockdown efficiency was assessed in normoxia and hypoxia (1% O_2_, 6 h). Sample loading was normalised with anti-β-actin staining. (**b**) SK-N-AS cells were treated with indicated concentrations of digoxin. The effect of digoxin on HIF-1α protein levels was assessed in normoxia or hypoxia (1% O_2_, 6 h). Sample loading was normalised with anti-β-actin staining. (**c**) Survival of SK-N-AS cells upon digoxin treatment. SK-N-AS cells were treated with indicated concentrations of digoxin and subsequently cultured under normoxia or hypoxia for 3 days. Cell viability was measured by MTS assay and is displayed relative to the control (DMSO-treated cells). (**d**) SK-N-AS-EGFP cells transduced with HIF-1β (upper panel) or SK-N-AS-EGFP treated with 10 nm digoxin for 3 days (lower panel) were implanted on the CAM of E7 chick embryos. Tumourigenesis on the CAM was assessed at E14 in at least 15 embryos. Representative images are shown. Scale bar is 500 μm. (**e**) Same as in (**d**) but cells were precultured in 1% O_2_ for 3 days. No metastasis could be detected in any of the embryos where primary tumours were found. Scale bar is 500 μm. (**f**) shHIF-1β SK-N-AS-EGFP or SK-N-AS-EGFP treated with 10 nm digoxin for 24 h were cultured for 3 days in 21% O_2_ or 1% O_2_ *in vitro*. mRNA levels relative to cyclophilin A were measured by qPCR. Bar graph represents the mean±s.e.m. of at least three independent experiments. *P⩽0.05, **P⩽0.01 and ***P⩽0.001 compared with normoxia. (**g**) The same cells and culture conditions as in (**f**) were used and implanted onto the CAM at E7. Three tumours from shHIF-1β cells and two from digoxin-treated cells were collected at E14 and mRNA levels relative to cyclophilin A were measured by qPCR. Bar graph represents the mean±s.e.m. (**h** and **i**) Same samples and results as in (**f** and **g**) but normalised to non-treated WT 21 and 1% cells, to demonstrate the difference in metastatic gene expression compared with WT cells (shown in Figure 5). Bar graph represents the logarithm of the normalised mean±s.e.m. of at least two independent experiments. ^#^*P*⩽0.05, ^##^*P*⩽0.01 and ^###^*P*⩽0.001 compared with WT cells cultured in 21% O_2_ and *P⩽0.05, **P⩽0.01 and ***P⩽0.001 compared with WT cells cultured in 1% O_2_.

Given the results obtained with DMOG, we aimed to probe the role of HIF activity in the metastatic phenotype and gene expression regulation. We used both a genetic and pharmacological approach to inhibit HIF-dependent transcription. An HIF-1β knockdown strategy was used to test a global HIF-1α/HIF-2α contribution, by blocking both HIF-1α/HIF-1β and HIF-2α/HIF-1β heterodimer formation needed for gene transcription regulation.^21^ HIF-1β expression was reduced by ~50% in shHIF-1β cells (Figure 6a). Alternatively, we used digoxin, a cardiac glycoside reported to inhibit translation of HIF-1α and HIF-2α mRNA.^22^ Digoxin treatment with 5, 10 or 100 nm resulted in a reduction of HIF-1α expression by 50%, 72% or 82%, respectively (Figure 6b). Digoxin also decreased cell survival by about 70% (Figure 6c). We selected 10 nm digoxin, which was sufficient to reduce HIF-1α levels by 72% in SK-N-AS cells exposed to 1% O_2_ while maintaining a survival of about 30% in normoxia and hypoxia.

Normoxic preculture of shHIF-1β-SK-N-AS- or digoxin-treated SK-N-AS cells resulted in tumourigenesis only for shHIF-1β cells (in 50% of chick embryos, similarly to WT cells) (Figure 6d). Tumours derived from shHIF-1β cells displayed deregulated vasculature and an almost complete lack of blood vessels. When cells were preconditioned in hypoxia, tumour formation was observed in both cell types (46% for shHIF-1β cells and 40% for digoxin-treated cells; Figure 6e). However, no metastasis was observed in either condition, suggesting the necessity of an active HIF module for the initiation of metastasis. At the transcriptional level of a selected subset of genes, shHIF-1β cells still showed an induction of HIF-1α target genes in hypoxia, *in vitro* (Figure 6f). On the contrary, *in vivo*, none of the HIF target genes nor classical metastatic genes were induced (Figure 6g), consistent with the avascular phenotype of the tumours. Digoxin blocked HIF-dependent transcription both *in vitro* and *in vivo* (Figures 6f and g). The induced transcription of HIF target genes in case of HIF-1β knockdown could be due to the remaining HIF-1β or compensation by HIF-2β subunits as has been reported for other NB cells.^23^ When mRNA levels measured in shHIF-1β or digoxin-treated cells were normalised to their counterpart WT untreated cells (Figures 6h and i), all genes promoting metastatic dissemination were significantly reduced both *in vitro* and in tumours, in line with the absence of invasion. These genes could therefore be used as markers to predict the cell's ability to metastasise. In summary, functional HIF transcriptional activity is essential for the metastatic phenotype of NB cells.

## Discussion

### Monitoring metastasis over time *in vivo*

The chick embryo model has proven to be a powerful tool, which can be readily imaged over time and in which the metastatic potential of NB cells and other cancer cells (for example, glioblastoma—unpublished observations) can be manipulated by a hypoxic preconditioning. This model is also compliant with the reduction of animal laboratory testing. Furthermore, it is particularly well adapted for NB, a tumour originating from neural crest cells during embryonic development, by mimicking its pathophysiological conditions.^24^ This was confirmed by the fact that the tumourigenesis observed (Figure 1 and Supplementary Figure S1) was similar to the clinical phenotype, including haemorrhagic tumours^18^ and metastasis in similar organs.^8, 19^ Tumourigenesis occurred frequently for both MYCN-amplified and -non-amplified NB cell lines, whereas spontaneous metastasis only occurred for cells precultured in hypoxia. Although our findings of tumourigenesis of SK-N-AS cells are in agreement with previous observations,^25^ the same group described spontaneous lung metastasis of SK-N-AS cells detected by qPCR of the human Alu sequence. No direct evidence of tumour growth in a given tissue was provided, thus it is possible that their findings originated from non-viable cell traces or artefacts. Here, fluorescent cell labelling enabled us to quickly and precisely visualise the location and incidence of metastasis.

### The impact of HIF-dependent transcription on cell survival and cell adhesion properties

We showed that hypoxic preconditioning strongly reduced *CASP8* and *DCC* expression, especially *in vivo*, potentially contributing to the aggressive phenotype. CASP8 and DCC have been associated with increased aggressiveness and poor prognosis in several cancers including NB,^25, 26, 27, 28^ and in NB lack of CASP8 has been found to promote metastasis.^29^

The increased occurrence of hypoxic preconditioned cells in proximity to blood vessels (Figure 3) indicates low binding properties to the primary tumour and epithelial–mesenchymal transition. This is supported by the regulation of *MMP2 and 9*, *TWIST1*, *SNAI1 and 2*, *CDH1*, and so on. Conversely, hypoxia resulted in a twofold decrease of velocity within the blood vessels, likely increasing their attachment to the endothelial vessel wall and extravasation. This can be explained by the hypoxic regulation of members of the Ig-CAM superfamily such as *VCAM*, *ICAM* and *NCAM*, as well as integrins (Figure 5), which all facilitate firm adhesion and transmigration. This complexity and difference in adhesion and migration pattern observed *in vivo* can explain the contradictory findings previously reported in *in vitro* studies using wound healing assays,^30, 31, 32, 33^ which provide poor representation of the multifaceted migration found in three-dimensional environments or *in vivo*.

The gene expression was long lasting, as shown by our qPCR experiments, with many genes showing very stable expression even after a relatively long reoxygenation period (Figure 5). This could be explained by the fact that a chronic hypoxic exposure allows long-lasting epigenetic remodelling,^34^ underlying the striking memory of the preexposure to hypoxia.

The observed changes in gene expression upon hypoxic exposure might not all be due to a direct HIF regulation, yet most of them have previously been shown to be regulated by either HIF-1α and/or HIF-2α. Surprisingly, we found that hypoxic preconditioning had no influence on HIF protein levels in the tumours (Supplementary Figure S3B), suggesting that HIF target gene expression rather than HIF levels is likely to provide more reliable markers to monitor tumour hypoxia or hypoxic episodes.

### Spatial and temporal features of hypoxic regions mediate metastatic progression

Malignant tumours experience a highly dynamic range of oxygen gradients,^10^ which is likely to impact on the spatial heterogeneity of tumours, forming a mixed population of normoxic and hypoxic cells. Remarkably, we observed that invasion by non-metastatic normoxic cells could be triggered by direct contact with hypoxic preconditioned cells (Figure 2). We propose the possible and non-exclusive following mechanisms: (1) hypoxic preconditioned cells prepare an invasive path by promoting matrix degradation or collective cell migration,^35^ which is supported by the fact that normoxic cells were only found in organs invaded by hypoxic cells. (2) Hypoxic and normoxic preconditioned cells could communicate via the transfer of microvesicles potentially containing mRNA, DNA or lipids.^36, 37, 38^ Indeed, several reports document that cancer cells under stress, such as hypoxia, shed vesicles,^38, 39^ which have been shown to influence their microenvironment-promoting metastasis.^36, 38^

In conclusion, we have here demonstrated that some hypoxic-induced phenotypes (including increased proliferation, target gene expression and migration properties) were missed in the *in vitro* culture system, which could have led to major misinterpretation if not combined with *in vivo* studies. Our results exemplify the importance of taking into account the spatial and temporal heterogeneity found in solid tumours and crucially demonstrate that the cells' 'history' within the tumour is of prime importance in understanding their phenotype. These findings could have major therapeutic implications in the development of cancer agents targeting hypoxic cells, and clearly highlight the complexity of targeting cancer cells at a given time in a constantly changing tumour.

## Materials and methods

### Cell culture

The human NB lines SK-N-AS and SK-N-BE(2)C (ECACC Nos 94092302 and 95011817) were grown in minimal essential medium supplemented with 10% foetal calf serum and 1% non-essential amino acids (both Life Technologies, Carlsbad, CA, USA) and maintained in a humidified incubator at 37 °C with 5% CO_2_. For hypoxic studies, cells were maintained at 37 °C with 5% CO_2_ and 1% O_2_ (Don Whitley Scientific, Shipley, UK; Hypoxystation-H35) or 8% (Eppendorf, Hamburg, Germany; Galaxy 48 R). For DMOG treatment, cells were cultured in media supplemented with 0.5 mm DMOG (Enzo Laboratories, Farmingdale, NY, USA) for 24 h. For digoxin treatment, 5, 10 or 100 nm digoxin (Sigma-Aldrich, St Louis, MO, USA) were added to the cells on day 0 followed by incubation as indicated.

### Stable cell line generation

Lentiviral particles were produced with the transfer vectors pHIV-dTomato (Addgene, Cambridge, MA, USA; plasmid 21374), pLNT-SFFV-EGFP^24^ and shHIF-1β (MISSION shRNA Plasmid DNA; Sigma-Aldrich) as described previously.^40^ Transduction efficiency of fluorescent cell lines was quantified by flow cytometry using a FACSCalibur Cytometer (BD Biosciences, Franklin Lakes, NJ, USA), while shHIF-1β cells were selected using puromycin (Gibco, Life Technologies).

### Survival assay

Cell survival assays were performed using CellTiter 96 AQueous One Solution Cell Proliferation Assay (MTS; Promega, Madison, WI, USA). To test for digoxin-induced cytotoxicity, 5 × 10^4^ cells per well were seeded in a 96-well plate, treated as indicated and analysed 3 days later according to the manufacturer's instruction.

### Immunocytochemistry and immunohistochemistry

For immunocytochemistry, cells were washed in phosphate-buffered saline and fixed in 4% formaldehyde (Sigma-Aldrich) for 10 min. Immunocytochemistry was performed using standard protocols. For immunohistochemistry (IHC), tissue samples were fixed for up to 12 h in 10% formalin (Sigma-Aldrich) or 4% formaldehyde for the preparation of paraffin or frozen sections, respectively. IHC of 4-μm-thick paraffin sections or 10-μm-thick frozen sections was performed using standard protocols.

Cell nuclei were stained with TO-PRO-3 (Life Technologies) and the following primary antibodies were used: mouse anti-Ki-67 (immunocytochemistry and IHC 1:100), mouse anti-CD56 (IHC-P 1:50; both Leica Novacastra, Wetzlar, Germany), rabbit anti-smooth muscle actin α (IHC-F 1:200), rabbit anti-MMP9 (IHC-P 1:100), rabbit anti-CA9 (IHC-P 1:1000; all Abcam, Cambridge, MA, USA), mouse anti-HIF-1α (IHC-P 1:100; NovusBio, Littleton, CO, USA) and rabbit anti-HIF-2α (IHC-P 1:1000; BD Biosciences). Antibodies used for IHC-P were validated and optimised in tissues known to show a positive reaction according to the human protein atlas (http://www.proteinatlas.org/). Cells were analysed by confocal microscopy (Zeiss, Oberkochen, Germany; lsm710) and images were acquired using the ZEN2012 software. Image analysis and cell quantification were performed with ImageJ (WS Rasband, ImageJ, U. S. National Institutes of Health, Bethesda, MD, USA). For quantification, a total of 500 to 1500 positive cells per slide and at least five slides were counted per experiment and condition.

### Quantitative PCR

For cells in culture qPCR was performed as described previously.^41^ A list of all primers used is provided in Supplementary Table S1.

For primary tissues, tumours harvested from the CAM were rinsed in ice-cold phosphate-buffered saline, transferred into RNAlater solution (Ambion, Life Technologies), isolated with NucleoSpin RNA Tissue Kit (Macherey-Nagel, Dueren, Germany) according to manufacturer's instructions and processed using the same methods described for cells.

### Western blot

Immunoblotting was performed as described previously^41^ with primary antibodies against HIF-1α (1:1000; BD Biosciences), HIF-1β (1:500; Novus Bio) or β-actin (1:1000; Abcam).

### Chick embryos

#### Intravenous injection and *ex vivo* imaging

Intravenous injection at E3 was performed as described previously.^24^ For coinjection, NB cells cultured under different conditions were mixed before injection. Cells were either imaged *in ovo* and followed until E10 using a standard fluorescent stereo microscope (Leica M165-FC) before dissection or the embryo was transferred onto a 3.5 mm glass bottom dish (Greiner, Bio-One, Frickenhausen, Germany) immediately after injection and imaged for up to 30 h at 37 °C with an epifluorescent microscope (Axio ObserverZ1; Zeiss) equipped with a fast acquisition CMOS camera (Andor, Belfast, UK). Videos were taken with a x10 objective and 500 frames at 29 frames per s were acquired. Quantification of cells in the circulation was performed on six different embryos from four independent experiments. Blood vessels close to the heart with a pulsatile rhythmic flow were not taken into consideration to avoid artefacts in the velocity calculation. About 150 cells were analysed for each condition. Velocity was calculated by converting the number of frames necessary for a cell to cross the field of view to μm/s.

#### CAM assay

For CAM implantation at E7, fluorescent NB cells were harvested as above and 1 × 10^6^ cells per μl were resuspended in serum-free media. CAM implantation was achieved by transferring 2 μl of the cell suspension into the membrane fold created by careful laceration. For coimplantation, NB cells cultured under different conditions were mixed before implantation. After CAM implantation, eggs were incubated until E14 and imaged using a standard fluorescent stereo microscope (Leica M165-FC). Tumours grown upon the CAM were imaged from three different perspectives (dorsal, ventral and lateral) demonstrating the depth of tumour growth. Average tumour volume was calculated using *V*=(4/3)π × length × height × depth of at least three different tumours and repeated in at least four independent experiments. Following removal of primary tumours from the CAM, embryos were dissected. Organs were removed and tumour cells and/or metastatic deposits identified by fluorescence.

### Statistical analysis

Statistical significance was computed using Student's *t*-test using Origin-Pro 8.6 (OriginLab Corporation, Northampton, MA, USA).
