# Supplementary material for: Cellular memory of hypoxia elicits neuroblastoma metastasis and enables invasion by non-aggressive neighbouring cells
Source: Oncogenesis. 2015 Feb 9;4(2):e138–. doi: 10.1038/oncsis.2014.52 (PMC4338426; doi:10.1038/oncsis.2014.52)
Supplement: Supplementary Movie Legends [file oncsis201452x1.doc]

**Video S1**

SK-N-AS-EGFP cells (precultured in 21% O_2_, 3 d) were injected in the blood vessels of an E3 chick embryo. Video was taken 40 min post injection by live *ex ovo* imaging, with a 5x objective and 1000 frames at 29 frames/s were acquired. Video display has been accelerated to 10 fps.

**Video S2**

SK-N-AS-EGFP (precultured in 1% O_2_, 3 d) and dTomato cells (precultured in 21% O_2_, 3 d) were co-injected in the blood vessels of E3 chick embryos. Videos were taken 10 min post injection by live *ex ovo* imaging, taken with a 10x objective, and 170 frames at 29 frames/s were acquired with an acquisition difference of 500 ms between channels for SK-N-AS-EGFP cells and dTomato. The two channels were overlaid in the video for ease of display.
