# Supplementary figures and images for: Cellular memory of hypoxia elicits neuroblastoma metastasis and enables invasion by non-aggressive neighbouring cells
Source: Oncogenesis. 2015 Feb 9;4(2):e138–. doi: 10.1038/oncsis.2014.52 (PMC4338426; doi:10.1038/oncsis.2014.52)

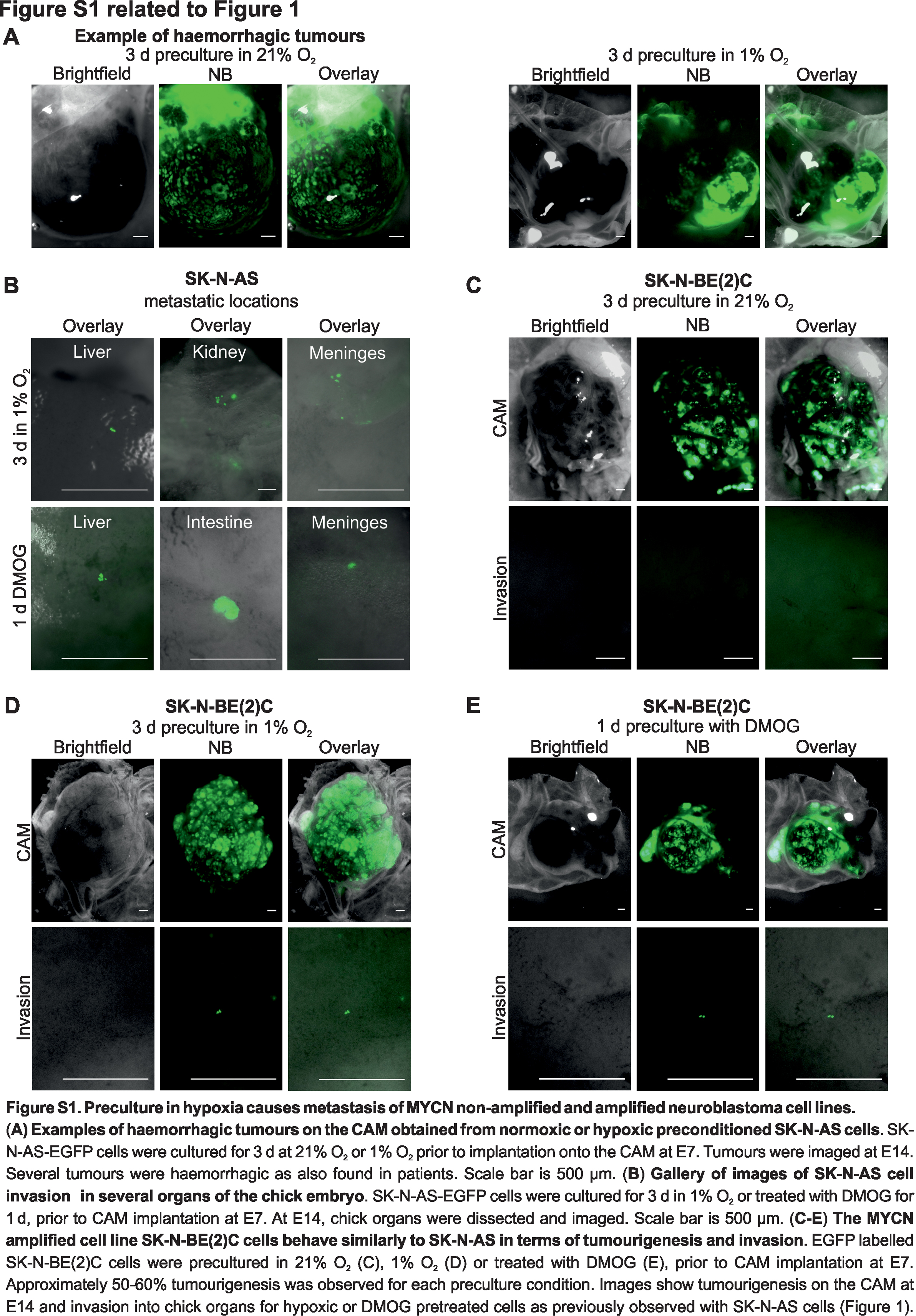

Supplement: Supplementary Figure S1 [file oncsis201452x4.tif]

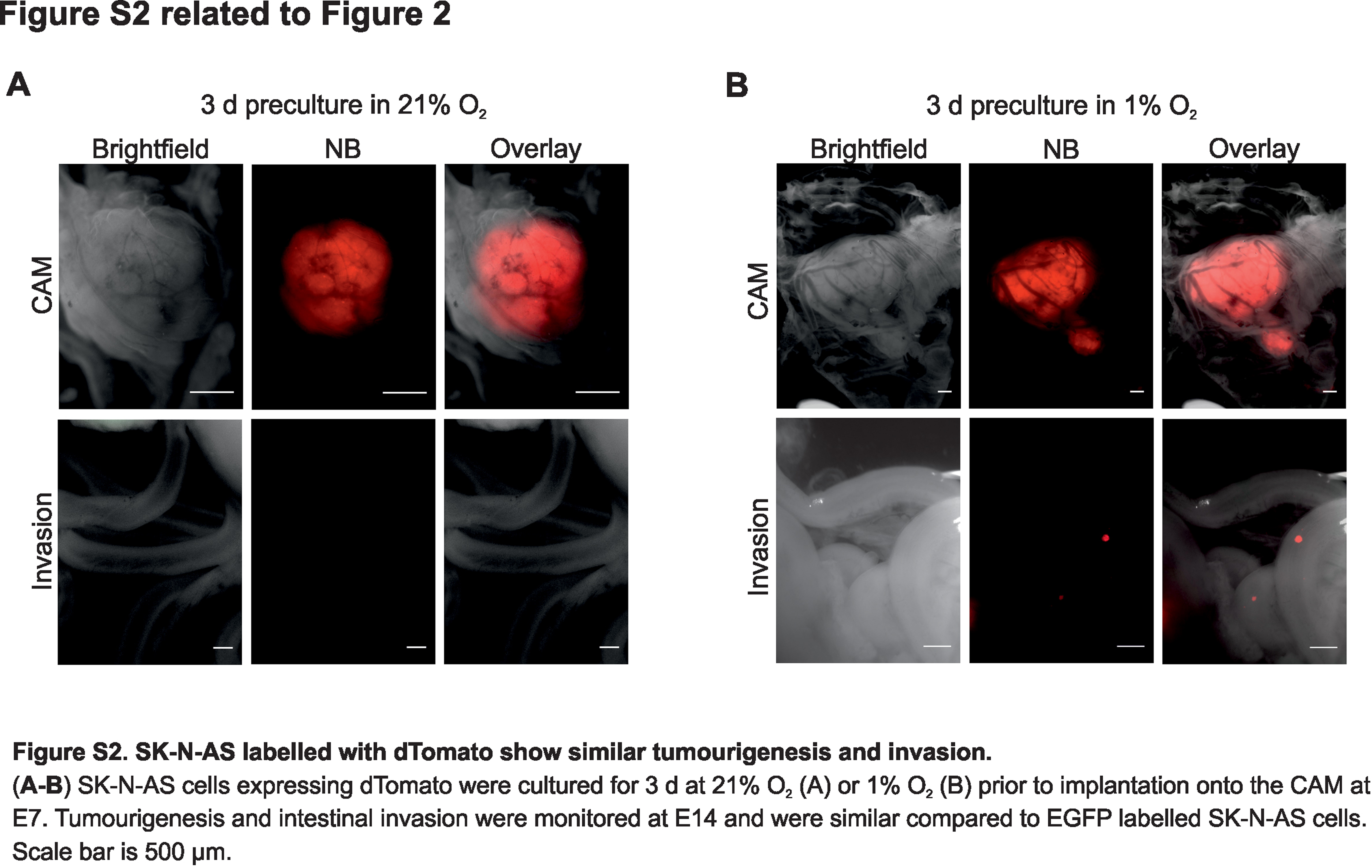

Supplement: Supplementary Figure S2 [file oncsis201452x5.tif]

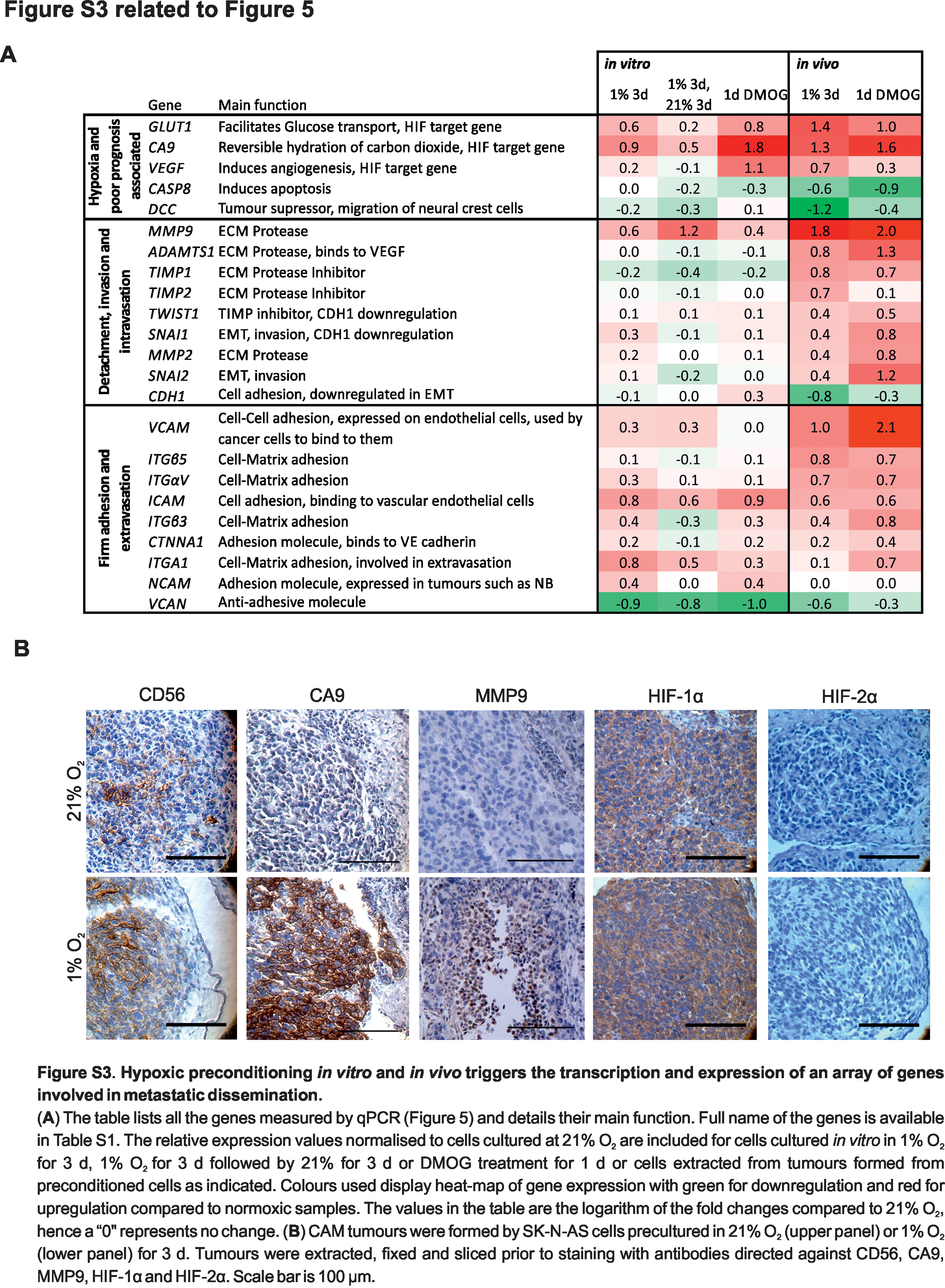

Supplement: Supplementary Figure S3 [file oncsis201452x6.tif]

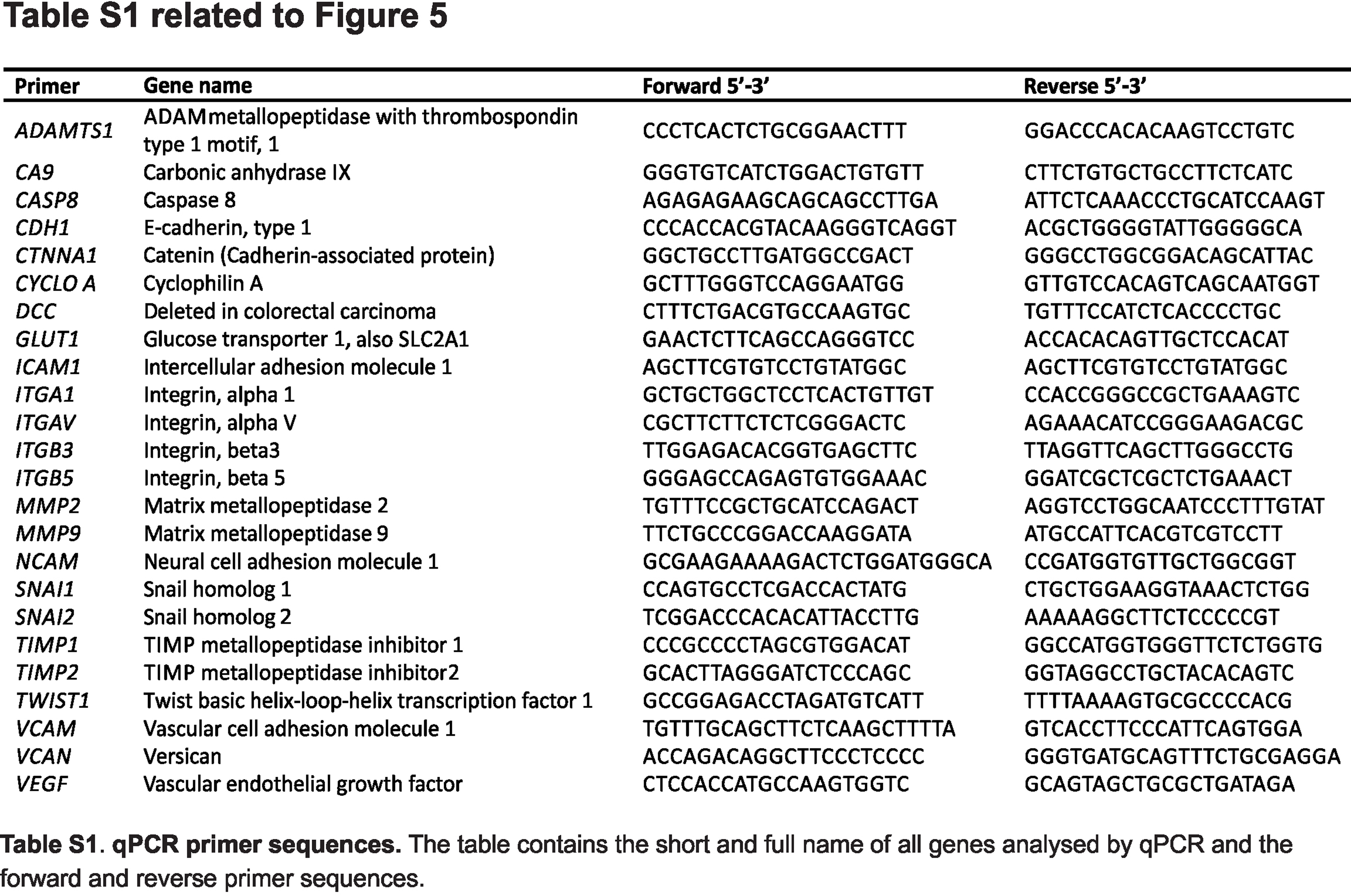

Supplement: Supplementary Table 1 [file oncsis201452x7.tif]
